# The British Neurosurgical Trainee Research Collaborative: Five years on

**DOI:** 10.1007/s00701-017-3351-5

**Published:** 2017-11-04

**Authors:** Aswin Chari, Aimun A. Jamjoom, Ellie Edlmann, Aminul I. Ahmed, Ian C. Coulter, Ruichong Ma, Paul May, Paul M. Brennan, Peter J. A. Hutchinson, Angelos G. Kolias, British Neurosurgical Trainee Research Collaborative

**Affiliations:** 1 Department of Neurosurgery, Royal London Hospital, London, UK; 2 Division of Neurosurgery, Department of Clinical Neuroscience, Western General Hospital, Edinburgh, UK; 3 Division of Neurosurgery, Department of Clinical Neurosciences, Addenbrooke’s Hospital and University of Cambridge, Cambridge, CB2 0QQ UK; 4 Department of Neurosurgery, Wessex Neurological Centre and University of Southampton, Southampton, UK; 5 Department of Neurosurgery, Royal Victoria Infirmary, Newcastle, UK; 6 Department of Neurosurgery, John Radcliffe Hospital, Oxford, UK; 7 Department of Neurosurgery, The Walton Centre, Liverpool, UK; 8 Surgery Theme, Cambridge Clinical Trials Unit, Cambridge Biomedical Campus, Cambridge, UK

**Keywords:** Collaborative, Research, Clinical trials, Resident, Trainee

## Abstract

Since its inception in 2012, the British Neurosurgical Trainee Research Collaborative (BNTRC) has established itself as a robust example of a trainee-led research collaborative. This article summarises the work of the collaborative over its first 5 years of existence, outlining the structure, its research projects, impact and future directions.

## Introduction

Multi-centre research can limit bias and can increase the external validity of study results. The British Neurosurgical Trainee Research Collaborative (BNTRC) was founded in 2012, with the aim of encouraging high-quality, multi-centre research and fostering a culture of collaboration. This includes engaging different units, increasing the accessibility of research for neurosurgical trainees and consultants and, ultimately, improving care and outcomes for neurosurgical patients. It was established as an initiative of the British Neurosurgical Trainee Association (BNTA) with the strong support of the Society of British Neurological Surgeons (SBNS) [14].

The BNTRC was inspired by the successes of the West Midlands Research Collaborative (WMRC), a regional general surgical research collaborative established 4 years prior. The WMRC had demonstrated significant success in implementing a trainee-led collaborative, including funding and running a randomised clinical trial that recruited to its target ahead of schedule [23].

The inaugural meeting was held at the Royal College of Surgeons of England (RCS) on 19 October 2012, with trainees from 13 of the 18 training programmes in the UK and Ireland in attendance and representatives from the BNTA and SBNS. Several projects were proposed by the attending trainees and a decision was made to pursue three projects (Table 1). Specific focus was placed on the national audit of chronic subdural haematoma (CSDH) as it was a topic relevant to trainees, and the prospective observational study model would serve as a proof of principle in terms of establishing the BNTRC network. Following the meeting, the SBNS agreed to support the BNTRC with a £10,000 starter grant to help with expenses [15].

**Table 1:** Studies selected for implementation at the inaugural BNTRC meeting in October 2012

Studies proposed at the inaugural BNTRC meeting
A national audit of chronic subdural haematoma (CSDH)
A national audit of cranioplasty
A randomised trial of craniotomy vs craniectomy for acute subdural haematoma (RESCUE-ASDH)

## Structure and research model

**Fig. 1:**
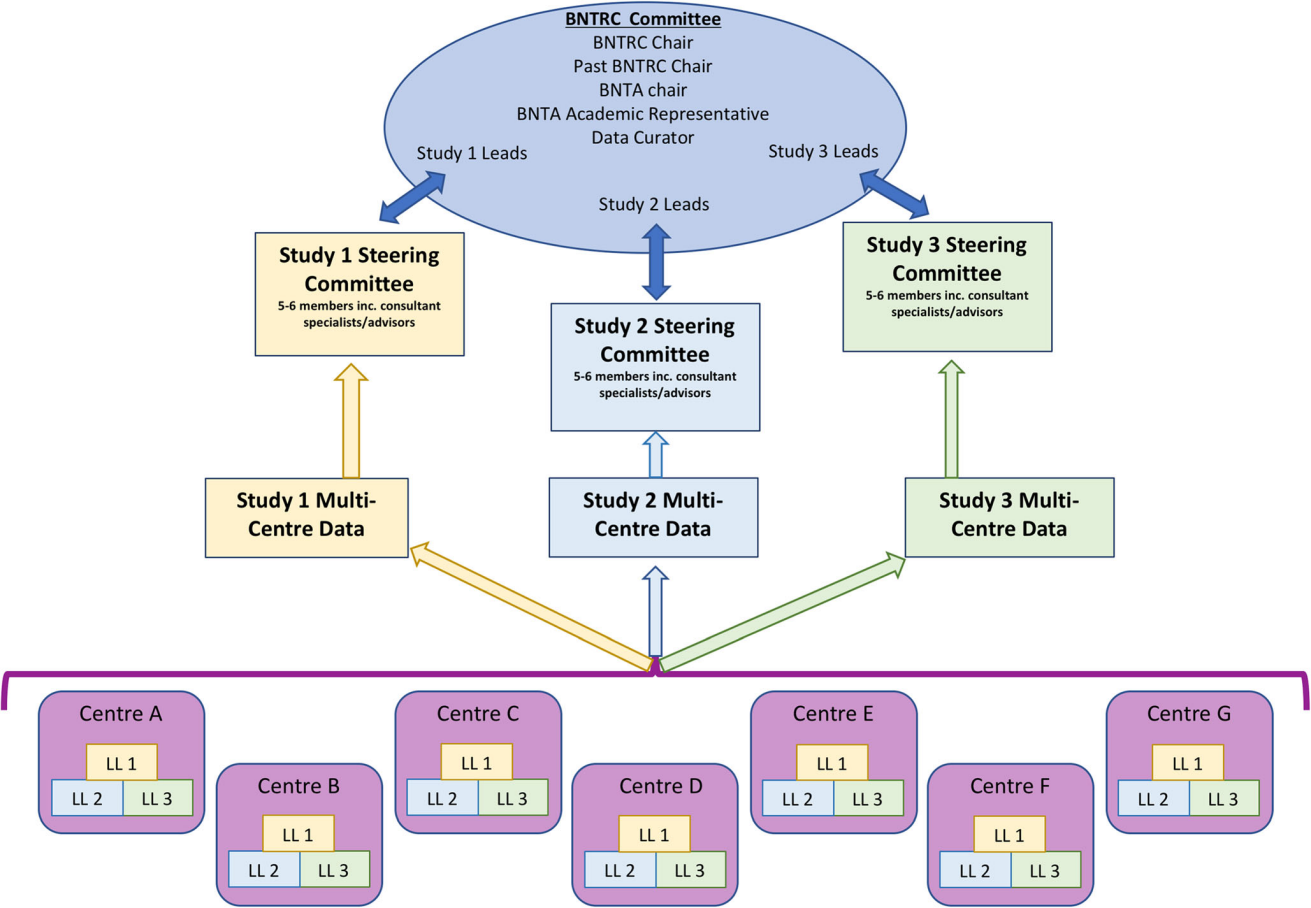
Collaborative research model used by the BNTRC. The BNTRC committee is composed of the individual study leads and representatives from the BNTA. Each study has a study steering committee and local leads (*LL*) at each neurosurgical centre

The formal structure of the BNTRC was established at an open meeting in January 2015, where a constitution was established [4]. Membership was opened to all junior doctors working in UK neurosurgery and the committee structure, involving the study leads and representatives from the BNTA, was upheld (Fig. 1).

The research model is based upon established trainee research collaborative models with each study having trainee leads (one to two members), a steering committee (five to six members) and local collaborators at each unit [1, 2]. The steering committee, which comprises of trainees and consultants, has responsibility for study design, execution, data integrity, analysis and write-up, with local collaborators being responsible for accurate data collection within each unit (Fig. 1). Although a general model, this is adapted for each study and is agreed in advance, in line with the constitution.

Protocols for each study are written by the study leads with input from the steering committee and are published (either online on our website or in a peer-reviewed journal) prior to study commencement. Each local lead is responsible for gaining relevant regulatory approvals (local audit, clinical governance or ethical approvals) and ensuring robust data collection. Once the data collection period is complete, the study leads and steering committee are responsible for data cleansing and analysis.

A key aspect of each study is the data management strategy. This includes finding robust platforms for data collection. The BNTRC has explored a number of strategies, from the simple method of combining anonymised data on Microsoft Excel to robust online platforms for data entry such as the Outcomes Registry Intervention and Operation Network (http://www.orioncloud.org) and Castor Electronic Data Capture (http://www.castoredc.com). Each have their advantages and disadvantages, and the best platform for each project must be individually chosen.

The prospectively agreed writing group, consisting of the study leads, steering committee and selected centre leads, are then responsible for drafting the manuscript, which is then sent to all collaborators prior to submission. Authorship criteria are broadly defined in the BNTRC constitution and each study steering committee also prospectively agrees the specifics of its own policy. The BNTRC is always the final author with all contributors acknowledged as PubMed-citable collaborators in published manuscripts. Following publication of the initial study manuscript, it was also agreed that the data would be made available to BNTRC members for further analyses, in a structured way [4].

## Research projects

Since 2012, the BNTRC has been involved with ten projects (four complete, six ongoing), with several new projects in the pipeline (Table 2). Project proposals are submitted through open calls to the entire UK neurosurgical trainee population (via established BNTA mailing lists). The proposals then undergo review by both the BNTRC committee and representatives of the SBNS academic committee, being assessed for the importance of the scientific question and the relative benefit of running a multi-centre study via the BNTRC. Whilst we initially started focusing on one project at a time, the collaborative has progressed to running multiple simultaneous projects.

**Table 2:** List of BNTRC projects of the past, present and future

Study name	Description	Project start	Project end	No. of collaborators	No. of neurosurgery trainees	No. of UK neurosurgical units	No. of patients	No. of conference presentations	No. of publications
National CSDH Audit	Prospective observational cohort study of management and outcomes of chronic subdural haematoma (CSDH).	2013	2015	78	52	26	1,205 patients	9	3
National EVD Audit	Prospective observational cohort study of practice patterns and infection rates of external ventricular drains (EVDs).	2014	2016	78	45	21	682 EVDs	8	2
RESCUE-ASDH trial	Randomised controlled trial of craniotomy vs decompressive craniectomy for acute subdural haematoma.	2014	On-going	84	39	26 (+ 9 international units)	525 (267 randomised and 258 observational) patients ADD *	Conference presentations for RESCUE-ASDH - 5	–
UK Cranial Reconstruction Registry (UKCRR)	Prospective observational study of cranioplasties to improve patient outcomes.	2015	On-going	TBC		TBC	–	1	1
Dex-CSDH trial	Randomised controlled trial of dexamethasone vs placebo for chronic subdural haematoma.	2015	On-going	54	19	19	459 patients ADD *	5	–
Angiogram-negative SAH Systematic Review	Systematic review to assess outcomes, complications, delayed investigations and diagnoses in subarachnoid haemorrhage (SAH) patients with negative initial neurovascular imaging.	2016	On-going	17	12	NA	–	4	–
RED-GB Audit	Prospective observational cohort study to evaluate practice patterns in patients scheduled for gross total resection of glioblastoma and the management of residual enhancing disease (RED).	2016	2017	34	18	15	113 patients	3	1
NANSIG Cauda Equina Syndrome Audit (ENTICE Study)	Collaborative project run by the Neurology and Neurosurgery Student Interest Group (NANSIG) and supported by the BNTRC. Retrospective study looking at adherence of cauda equina syndrome referrals to published national guidelines.	2017	2017		28	26 (+ 3 spinal units)	3,850 patients	–	–
Cauda Equina Syndrome Observational Study	Prospective observational study of the referral patterns, management and outcomes of patients with cauda equina syndrome.	2017	In set-up	Inviting collaborators		Inviting units	–	–	–
Cerebral Metastases Observational Study	Prospective observational study of the referral patterns, management and outcomes of patients with cerebral metastases.	2017	In set-up	Inviting collaborators		Inviting units	–	–	–

References for the publications: [3, 5, 8, 9, 12, 13, 17]

* number of patients as of 15/10/2017

The two randomised controlled trials, the Dex-CSDH and Rescue-ASDH trials (Table 2), have attracted funding from the UK National Institute of Health Research (NIHR) and are supported by the Cambridge Clinical Trials Unit. Although run by consultant-level investigators, the BNTRC and its members have had a crucial role in protocol development, funding applications and continue to play vital roles in patient recruitment and trial delivery. Collectively, these studies have randomised more than 600 patients in the last 3 years, illustrating the feasibility of trainee collaborative-led trial delivery.

## Communications, website and social media

Communication and raising awareness about the BNTRC and its projects is a central strategy of the collaborative. To achieve this, the BNTRC has developed a website, produces a regular newsletter and is active on social media outlets such as Twitter.

In a bid to be as inclusive as possible, the BNTRC sends all communications via the established BNTA mailing list, which is open to all non-consultant doctors working in neurosurgery in the UK. Additional communications (such as newsletters) are distributed more widely, including to the SBNS and European Association of Neurosurgical Societies (EANS).

A BNTRC website was also developed to act as a resource for trainees and interested parties to learn more about the collaborative. It contains a wealth of information on the structure of the group, its constitution and the range of BNTRC projects. It is an important route for communication from interested doctors and medical students. Regular communications are also sent out via the BNTRC Twitter account (@BNTRC), which acts as an important social media resource for the collaborative. The group has an active account with over 650 followers and 9600 impressions over a 3-month period (Feb-Apr 2017). It is a useful means to communicate news and progress reports about BNTRC projects.

## Impact: benefits for all stakeholders

The most important stakeholders in collaborative research are patients, who stand to benefit through the comprehensive, high-quality investigation of clinically relevant questions. Over the last 5 years, the projects completed by the BNTRC have demonstrated the ability to benefit patients, in terms of identifying practices associated with improved outcomes. For example, the National CSDH audit identified that practices associated with unfavourable outcomes included lack of post-operative drain, use of post-operative bed rest and single burr-hole drainage [3].

Neurosurgical trainees also stand to benefit from involvement with the BNTRC. Trainees get the opportunity to be involved in the development and execution of robustly designed research protocols, skills that are vital in inculcating a generation of academically equipped neurosurgical consultants of the future. We also hope to contribute positively to the high-calibre research output of the UK neurosurgical community, which has produced many recent landmark clinical trials, including the STICH trials, RESCUEicp and STASH [7, 11, 18, 19].

From a national perspective, the BNTRC is viewed as one of the flagship specialty surgery research collaboratives, both in terms of the extent of its research activities and output so far [10, 20]. Importantly, the network has engaged trainees from all UK neurosurgical units (Table 2). It has also played a key role in the National Research Collaborative (NRC), the umbrella body for UK surgical research collaboratives. It co-hosted the 2016 NRC Meeting, at the Royal College of Surgeons of England (London) in November 2016, along with the Reconstructive Surgery Trials Network (RSTN), the plastic surgery collaborative. It has also played a key role in shaping NRC policy, in terms of recognising and rewarding collaborative research and bolstering the infrastructure of trainee-led research collaboratives [16].

As the first of its kind in the global neurosurgical community, the BNTRC has played a pivotal role in setting an example to trainee neurosurgeons around the world. The collaborative has served as an inspiration and has provided support to a number of other collaboratives, including the Canadian Neurosurgical Research Collaborative (CNRC) [24], the Dutch Neurosurgical Trainee Research Network (DNTRN) and a new Scandinavian collaborative that is at its inception.

## Challenges

Over the last 5 years, the collaborative has faced a number of challenges. We have addressed some of these in this section, which, along with a previously published ‘how to’ guide [6], should act as a useful resource to trainees looking to establish their own research collaboratives.

At inception, the biggest challenges include selling the model to trainees, consultants and supporting professional bodies. The BNTRC has been fortunate in having the unwavering support of the SBNS and the SBNS academic committee. Examples of successful trainee research collaboratives, such as the WMRC, were instrumental in convincing the trainee community of the potential of the collaborative research model.

The choice and success of the first project is also crucial to convincing all the stakeholders of the potential of the collaborative. It is prudent to pick a simple, non-controversial research question, with an observational study design that will serve as a conduit for establishing the multi-centre network. The study should have a relatively short timescale, with clearly established author and collaborator policies, rewarding all collaborators for their involvement.

Each project also requires dedicated and motivated project leads and steering committee members, who are willing to drive the project forwards. Key challenges for the committee include developing a watertight protocol, obtaining ethical approvals and assembling the team of collaborators at each centre. In the UK, approvals are facilitated by having separate approval processes for ‘audit’ projects, which are less stringent than the ethical approval process for research projects. Data collection platforms also require careful thought, taking into account ease of use, financial considerations and security of the platform. As outlined above, the BNTRC has used a number of different platforms and we feel this flexibility has educated the group about the pros and cons of each, allowing us to tailor the choice of platform based on the particular needs of future projects.

Although the BNTRC established their constitution 3 years into its existence, we would advocate new groups establish a constitution at inception, outlining transparent policies for authorship, leadership and choice of new projects.

## Future directions

In addition to completing and continuing to expand the current portfolio of projects, there are several challenges that lie ahead. First and foremost is sustaining enthusiasm and interest in collaborative research, and this can only be achieved by ensuring that the collaborative projects are relevant to our specialty. Enhancing our relationship with the SBNS and each of the subspecialty groups (e.g. trauma, vascular, CSF, oncology, spinal, etc.) will facilitate this. Second, maintaining the highest standards when conducting audit and research will be critical. Following relevant guidance, such as the IDEAL framework, will be helpful in that respect. The IDEAL framework describes the research stages from first-in-human to evaluation in randomised trials (www.ideal-collaboration.net) [22]. Third, the collaborative projects rely on the voluntary contributions (time and effort) of trainees; this means that their contribution needs to be fairly recognised and rewarded. Moreover, as senior trainees qualify, a role for them should be found within the network, as their experience will be valuable. Fourth, trials and studies enrolling patients in the non-emergency setting are clearly important in neurosurgery and we believe that the BNTRC could be successful in that respect too. However, this assumption will need to be tested in the future as studies in the non-emergency setting have their own set of challenges (e.g. strong surgeon and patient preferences). Other specialty trainees, including neurologists, radiologists and anaesthetists should be engaged in cross-specialty projects that will be crucial to improving patient care too. Finally, trainee collaboration at a global level is an exciting opportunity for enhancing the relevance of research findings for even more patients. The BNTRC remains committed to assisting trainees interested in setting up collaboratives in their countries with practical advice and support. The trainee collaborative model, at a global level, can contribute to the infrastructure networks required to increase access to neurosurgical services in low- and middle-income countries [21].

## Conclusions

In the 5 years since its inception in 2012, the BNTRC has come a long way. There are significant challenges that lie ahead in sustaining and expanding upon this early success, with much of it depending on the sustained enthusiasm of future generations of UK neurosurgical trainees.
